# People’s desire to be in nature and how they experience it are partially heritable

**DOI:** 10.1371/journal.pbio.3001500

**Published:** 2022-02-03

**Authors:** Chia-chen Chang, Daniel T. C. Cox, Qiao Fan, Thi Phuong Le Nghiem, Claudia L. Y. Tan, Rachel Rui Ying Oh, Brenda B. Lin, Danielle F. Shanahan, Richard A. Fuller, Kevin J. Gaston, L. Roman Carrasco

**Affiliations:** 1 Department of Biological Sciences, National University of Singapore, Singapore; 2 Environment & Sustainability Institute, University of Exeter, Penryn, Cornwall, United Kingdom; 3 Centre for Quantitative Medicine, Duke-NUS Medical School, Singapore; 4 School of Biological Sciences, Centre for Biodiversity and Conservation Sciences, University of Queensland, Brisbane, Australia; 5 CSIRO Land & Water Flagship, Dutton Park, Queensland, Australia; 6 Centre for People and Nature, Zealandia Ecosanctuary, Wellington, New Zealand; 7 Victoria University of Wellington, Wellington, New Zealand; Oxford University, UNITED KINGDOM

## Abstract

Nature experiences have been linked to mental and physical health. Despite the importance of understanding what determines individual variation in nature experience, the role of genes has been overlooked. Here, using a twin design (TwinsUK, number of individuals = 2,306), we investigate the genetic and environmental contributions to a person’s nature orientation, opportunity (living in less urbanized areas), and different dimensions of nature experience (frequency and duration of public nature space visits and frequency and duration of garden visits). We estimate moderate heritability of nature orientation (46%) and nature experiences (48% for frequency of public nature space visits, 34% for frequency of garden visits, and 38% for duration of garden visits) and show their genetic components partially overlap. We also find that the environmental influences on nature experiences are moderated by the level of urbanization of the home district. Our study demonstrates genetic contributions to individuals’ nature experiences, opening a new dimension for the study of human–nature interactions.

## Abstract 2

Nature experiences have been linked to mental and physical health. This twin study reveals genetic influences on an individual’s orientation towards nature and nature experiences, opening a new dimension to understanding human-nature interactions.

## Introduction

In recent decades, human populations have shifted markedly from rural to urban environments, with more than 55% of people now living in cities [1]. While there are advantages to urbanization, urban living is, nonetheless, associated with poorer mental health, reduced subjective well-being, and a higher risk of psychiatric disorders [2–4]. A reduction in nature experiences in urban environments has been shown to be a key risk factor for mental health issues and is associated with an increased risk of anxiety and depression [5–7]. However, there is marked variation in the extent to which individuals within populations have nature experiences [8–10], and this inevitably affects who receives the associated physical and psychological benefits from experiencing nature [6].

The causes of variation in people’s experiences of nature include their opportunities to interact with nature and their orientation toward obtaining such experiences [8,11,12]. On the one hand, people living in places with more nature available (nature opportunity) tend to interact with nature more frequently [5,11,13–15]. On the other hand, people with a stronger desire to experience nature (nature orientation), with a higher willingness to travel farther to experience nature and to spend more time in gardens, are likely to gain more experiences of nature [5,11,14,16,17]. Nature orientation and opportunity are, however, not independent from each other [12]. For instance, individuals who have a strong nature orientation may choose not to live in highly urbanized areas [13]. Alternatively, nature opportunity may enhance a person’s nature orientation [12]. These cause–effect relationships with many possible causal connections are extremely hard for correlative studies to disentangle.

A person’s nature orientation and opportunity can be shaped by environmental [14,18–20] and genetic factors [21–23]. The genetic contribution to nature orientation has been hypothesized, for example, through the biophilia hypothesis [21], but has never been tested. If such a genetic contribution exists, we can test whether the positive correlation between nature orientation and nature experience could be shaped by a shared genetic basis. By considering the level of urbanization of an individual’s home location (as a proxy of nature opportunity) as a phenotype, we can also test whether there is a genetic component in the level of urbanization of people’s home location and whether it overlaps with the genetic basis of nature orientation (genetic niche picking). If an overlap exists, it may support the mechanism that people would (genetically) choose to live in rural/urban areas through their strong/weak nature orientation. However, as other socioeconomic factors may constrain individuals in their choice of home location, the level of urbanization also functions as an environmental factor influencing a person’s nature orientation and nature experience. Such an environmental factor (the level of urbanization) may also moderate the genetic effects on nature orientation and nature experience (i.e., through gene–environment interactions).

While the importance of combining genetic and environmental factors to understand human behavior is widely recognized [24], estimating the contribution of genes and environments, or their interactions, to individual variation in nature experience remains unexplored. Here, we estimate the extent to which genetic and environmental influences can explain individual variation in nature experience. Our research questions are the following: (i) are nature orientation, nature opportunity (the level of urbanization of home location), and nature experience heritable?; (ii) if heritable, are there shared genetic bases among these traits?; and (iii) are the genetic influences on nature orientation and nature experience moderated by the level of urbanization of home location?

We answer these questions using a twin approach that allows us to tease apart genetic and environmental influences. We use the TwinsUK panel [25] (number of twin individuals surveyed = 2,306) to examine the extent to which genetic versus environmental influences explain individual variation in nature orientation, the level of urbanization of home location (at the district level), and 4 dimensions of nature experience (frequency and duration of public nature space visits and frequency and duration of domestic garden visits). Based on the assumption of differences in the genetic similarity of monozygotic (MZ) twins (100%) and dizygotic (DZ) twins (50%), we partition phenotypic variance into additive genetic (A), shared environmental (C; shared between the twin pairs), and unique environmental influences (E; unique to each twin individual and including measurement error). After controlling for sex and age, we build a multivariate ACE model with a direct symmetric approach. The multivariate model allows us to examine the genetic and environmental correlations between phenotypes. We also build full bivariate moderation models to further estimate the effect of the level of urbanization of home district on the genetic and environmental influences on nature orientation and of 4 dimensions of nature experience. The full bivariate moderation model allows us to account for potential gene–environment and environment–environment correlations [26,27].

## Results

### Heritability and genetic/environmental correlations

**Fig 1:**
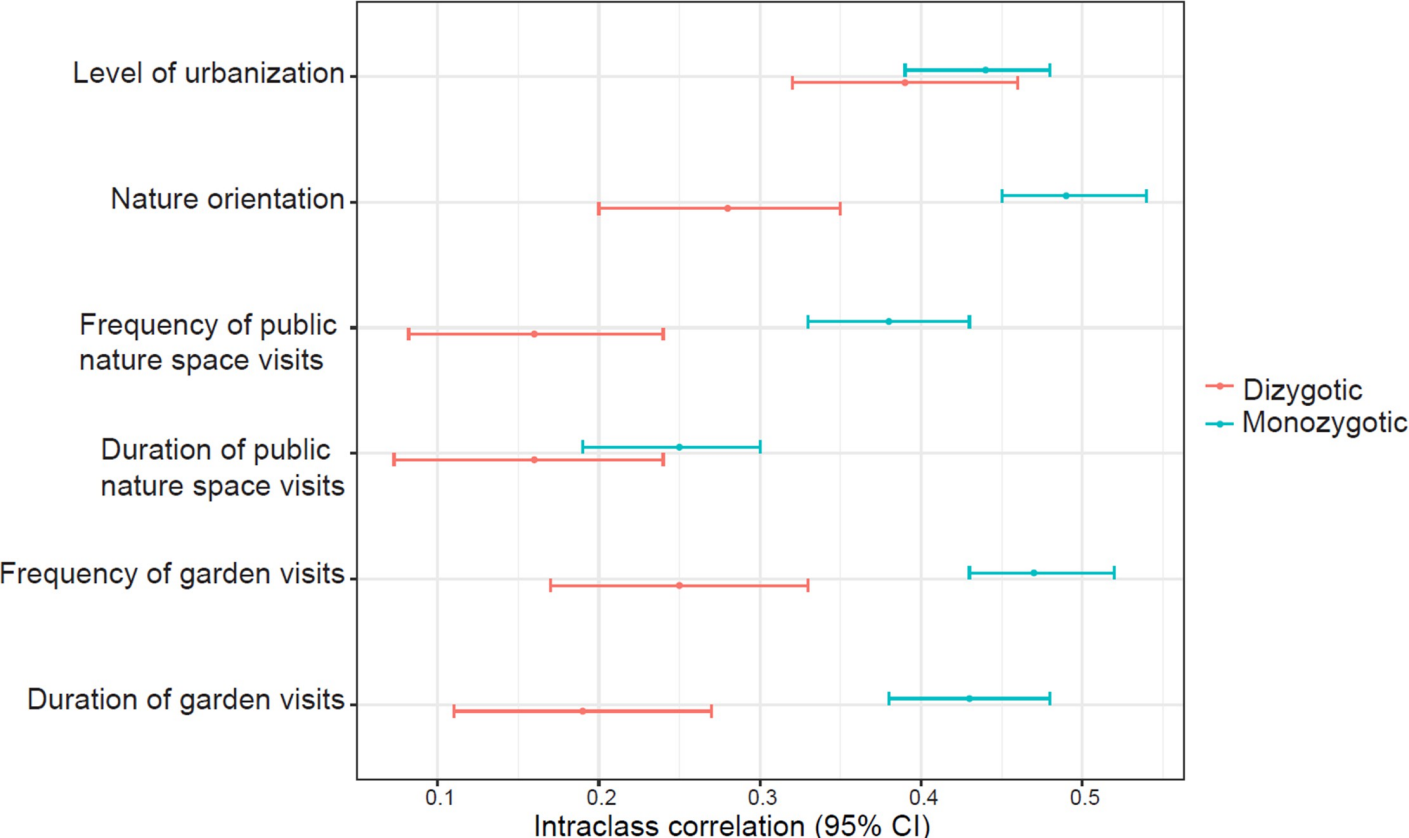
Intraclass correlation between MZ twin pairs and between DZ twin pairs. No overlap between MZ and DZ correlations implies genetic influences on the traits. The intraclass correlation with traits controlling for sex and age is included in S1 Table. CI, confidence interval; DZ, dizygotic; MZ, monozygotic. The data and code underlying this figure can be found at https://doi.org/10.6084/m9.figshare.17054540.v1.

MZ twins were more similar to each other in nature orientation and 3 of 4 dimensions of nature experience (frequency of public nature space visits and frequency and duration of garden visits) than DZ twin pairs (Fig 1). By contrast, the intraclass correlation between MZ pairs and that between DZ pairs on level of urbanization of their home district and on duration of public nature space visits partially overlapped (Fig 1), suggesting weak genetic influences.

**Fig 2:**
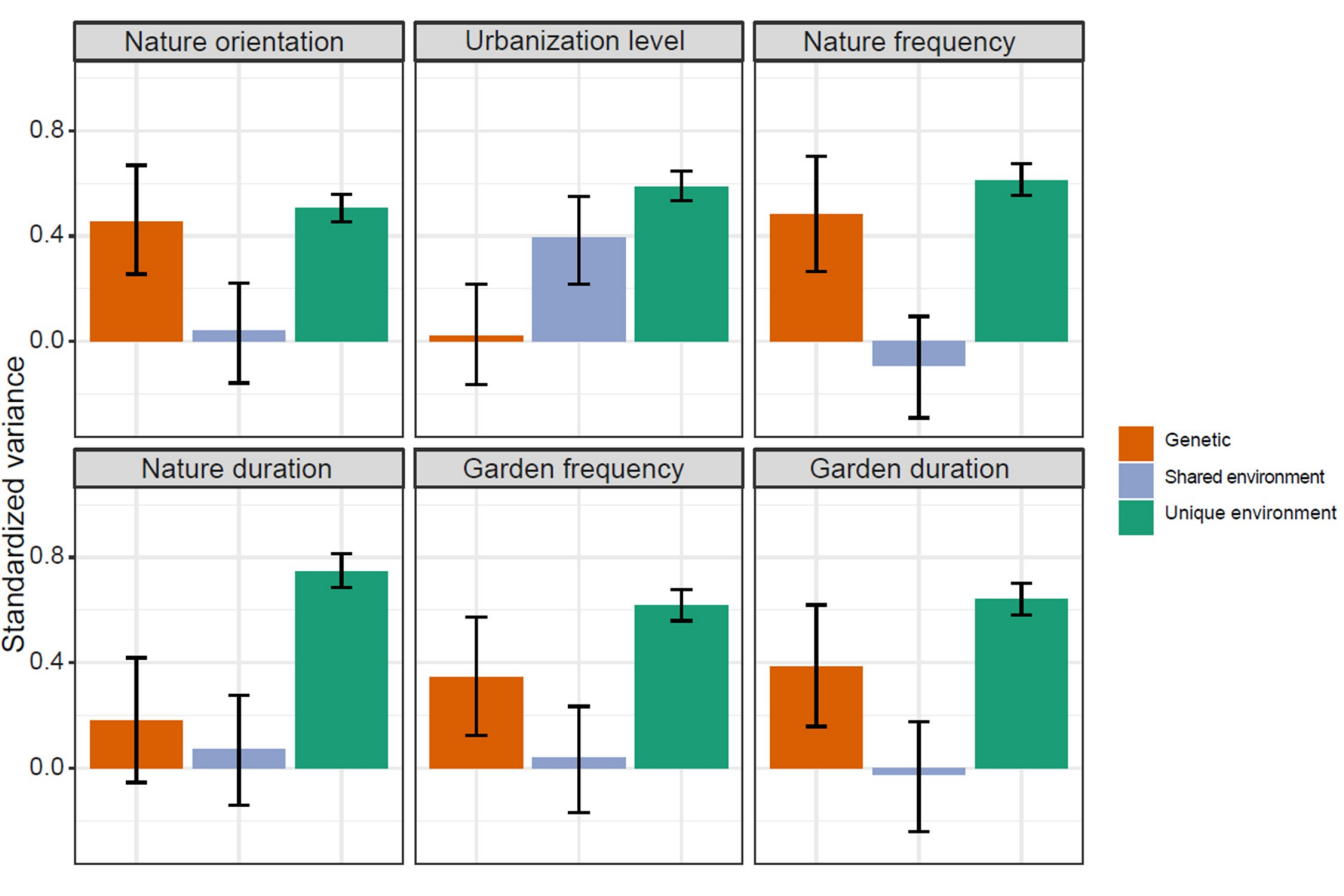
Genetic and environmental influences. Standardized genetic and environmental variance explained in nature orientation, level of urbanization of home district (urbanization level), frequency of public nature space visits (nature frequency), duration of public nature space visits (nature duration), frequency of garden visits (garden frequency), and duration of garden visits (garden duration) with the multivariate model controlling for sex and age using a direct symmetric approach. Error bars = 95% CIs. CI, confidence interval. The data and code underlying this figure can be found at https://doi.org/10.6084/m9.figshare.17054540.v1.

In our multivariate ACE model, nature orientation (heritability = 46%, 95% confidence interval (CI) = 26% to 67%), frequency of public nature space visits (heritability = 48%, 95% CI = 27% to 70%), frequency of garden visits (heritability = 34%, 95% CI = 13% to 57%), and duration of garden visits (heritability = 38%, 95% CI = 16% to 62%) were moderately heritable (Fig 2). There was negligible heritability for the level of urbanization of home district and duration of public nature space visits (Fig 2).

**Fig 3:**
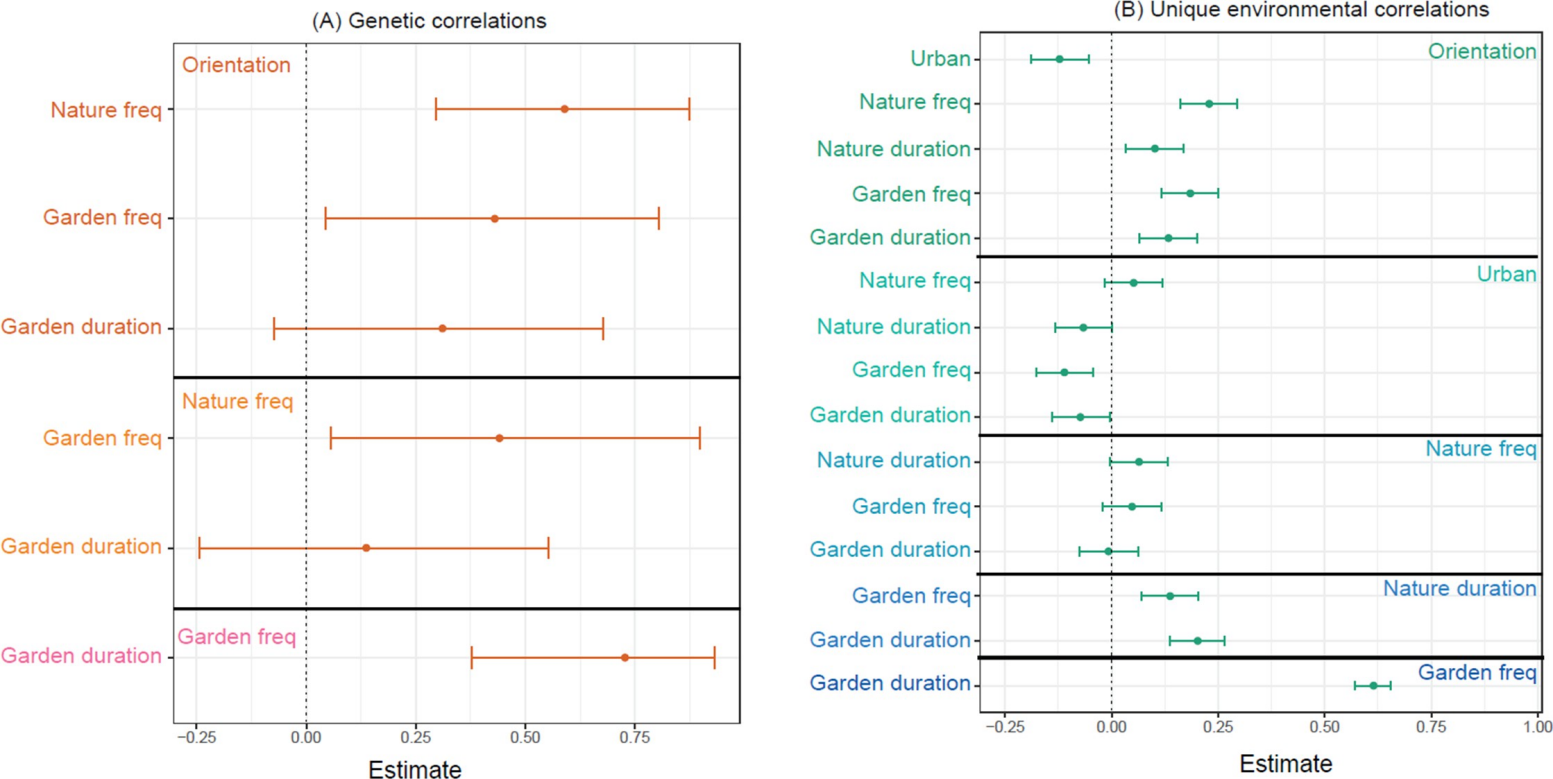
Genetic and unique environmental correlations. Estimates of **(A)** genetic and **(B)** unique environmental correlations of nature orientation (orientation), level of urbanization of home district (urban), frequency of public nature space visits (nature freq), duration of public nature space visits (nature duration), frequency of garden visits (garden freq), and duration of garden visits (garden duration) from the multivariate model controlling for sex and age using a direct symmetric approach. Error bars = 95% CIs. The genetic correlations were not estimated if there was negligible heritability (Fig 2, the CI of heritability includes negative values). CI, confidence interval. The data and code underlying this figure can be found at https://doi.org/10.6084/m9.figshare.17054540.v1.

There was a high positive genetic correlation between nature orientation and frequency of public nature space visits (0.59, 95% CI = 0.30 to 0.88, Fig 3A) and a moderate positive genetic correlation between nature orientation and frequency of garden visits (0.43, 95% CI = 0.04 to 0.80, Fig 3A). These results indicate that genetic components of individual variation in nature orientation and frequency of nature experiences are partially shared; a higher level of nature orientation may predispose individuals to visit parks and gardens more frequently or vice versa.

There were negligible shared environmental influences on nature orientation and 4 dimensions of nature experience (Fig 2), but a moderate shared environmental influence on the level of urbanization of the home district (39%, 95% CI = 22% to 55%, Fig 2). The unique environmental influences explained more than 50% of the individual variation in all 6 phenotypes (Fig 2). However, there were generally low unique environmental correlations among these phenotypes (Fig 3B, e.g., unique environmental correlations between nature orientation and frequency of public nature space visits = 0.23, 95% CI = 0.16 to 0.29). These results indicate that there may be different environmental factors influencing a person’s nature orientation, nature experiences, and whether a person lives in a rural/urban area.

### Moderation of the level of urbanization on genetic/environmental influences

**Fig 4:**
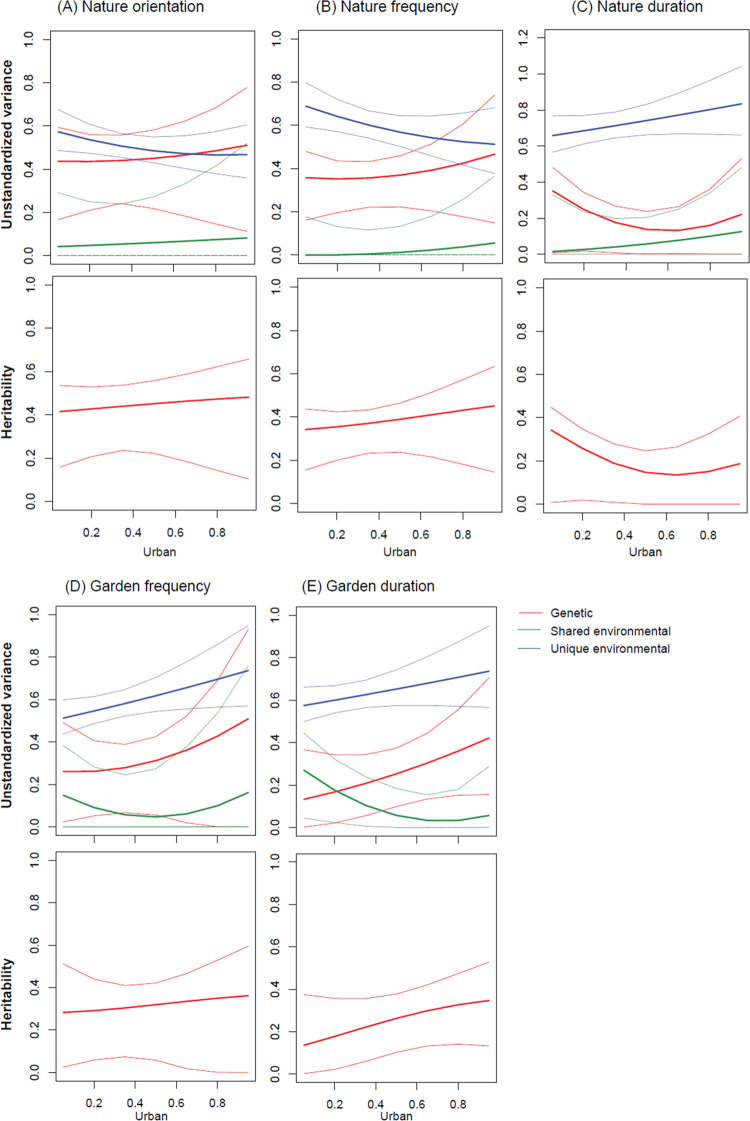
Moderation of the level of urbanization on the genetic and environmental influences. Unstandardized genetic and environmental variances (upper panels) and heritability (lower panels) in **(A)** nature orientation, **(B)** frequency of public nature space visits, **(C)** duration of public nature space visits, **(D)** frequency of garden visits, and **(E)** duration of garden visits as a function of level of urbanization of the home district (urban; 0 = rural areas, 1 = highly urbanized areas) based on the moderation models controlling for age and sex. The thick lines are the estimated variances at different levels of urbanization, and the thin lines are the 95% CIs. Model comparisons to test the statistical significance of the moderation effect are shown in S3 Table. CI, confidence interval. The data and code underlying this figure can be found at https://doi.org/10.6084/m9.figshare.17054540.v1.

Our moderation models tested whether the level of urbanization of the home district moderated the genetic and environmental influences on nature orientation (Fig 4A) and nature experiences (Fig 4B–4E). The path coefficients are shown in S2 Table, and model comparisons to test the significance of moderation effects are shown in S3 Table. The unique environmental influence on the frequency of public nature space visits was significantly reduced with increasing levels of urbanization (Fig 4B, S3 Table), but unique environmental influence on the frequency of garden visits was significantly increased with increasing levels of urbanization (Fig 4D, S3 Table). However, we did not detect any significant change in the genetic influences on nature orientation and nature experiences across the levels of urbanization (Fig 4, S3 Table).

## Discussion

The results demonstrate genetic contributions to nature orientation and nature experience, while environmental contributions are the predominant source of individual variation. We also find that the genetic basis of nature orientation and of frequency of nature experience partially overlap, as supported by positive genetic correlations. The environmental factors for nature orientation and those for nature experience may be different, with low environmental correlations, suggesting that diverse behavioral interventions or urban planning strategies might be necessary to increase the well-being benefits that people can gain from nature experiences. The environmental influences on frequency of nature experience were further moderated by the level of urbanization of the home district.

### Importance of genetic and environmental contributions

Our results shed light on the debate on the cause–effect relationship between nature orientation and nature experiences [11–13] by suggesting that their relationship is due to a partially shared genetic basis. One potential mechanistic explanation is that some genes underlying nature orientation (i.e., a stronger desire to seek nature experience) might drive a person to experience nature more frequently. Importantly, the role of nature orientation goes beyond simply visiting nature, and it may also mediate the amount of psychological well-being that a person gains from nature experiences. There is evidence that people who are more oriented toward nature can reap more psychological benefits from nature experiences, such as improved life satisfaction or mood [28–30]. However, unlike life satisfaction and mood, a high level of nature orientation may not be necessary for one to gain cognitive benefits from nature experiences [31,32]. Considering the genetic component of nature orientation, individuals with certain genetic variants may be more responsive to exposure to natural environments when it comes to their psychological well-being [33].

Despite the genetic influences, environmental factors explained more than half of individual variation in nature experiences. The environmental factors influencing nature experience may include, among others, the travel time and distance to the nearest nature space and the quantity and quality of nature that is accessible to the individual [14,15]. But these environmental factors for nature experience do not necessarily associate with those influencing nature orientation, as noted by their low but positive environmental correlations. The lack of a substantial overlap in environmental factors influencing nature experience and nature orientation highlights the importance of diverse urban planning in providing accessible and good quality natural spaces, along with separate interventions to strengthen an individual’s orientation toward experiencing nature.

We find very little genetic influence on the level of urbanization of home district, while about 40% of individual variation can be explained by the shared environmental factors. The shared environmental factors may respond to, for example, socioeconomic factors. For instance, socioeconomic status may correlate with access to nature [19,34–36]. Despite the commonly reported relationship between level of urbanization and nature experience [5,11,13–15], the unique environmental correlations between them were low. This may be because the level of urbanization was quantified at the district level due to confidentiality reasons, and people in the same district could differ markedly in their actual opportunity to visit nature spaces. The unique environmental correlation between the level of urbanization and public nature space visits may be higher if future studies have access to higher resolution spatial data.

### Influence of age on genetic and environmental contributions

A person’s nature orientation has been suggested to result from learning (i.e., through environmental factors) based on the observation that children prefer urban over natural environments, and such urban preference declines with age [32]. While Meidenbauer and colleagues documented the change of mean preference for natural environments across age, we focused on how individuals differ from each other by comparing the similarity between MZ twins and the similarity between DZ twins to estimate the sources of individual variation [32]. Our results are not necessarily at odds but complement findings in [32], since a learned behavior does not conflict with evidence of a behavior being heritable (e.g., academic performance is highly heritable in adequate environments [37]). Similarly, nature orientation could be partially heritable but requires the right environment for its expression.

Our study reveals a low shared environmental influence (such as parental family) on nature orientation. It is possible that this parental effect on nature orientation, as reported by [32], is only strong for children and teenagers, and we were unable to detect such effect as our study population comprised relatively old adults (mean = 60.4, ranging from 19 to 89). This seemingly opposing result could be explained by parental influence not lasting and declining once individuals leave the parental home [38]. Future studies performing twin analyses on nature orientation using children or teenagers could help complement our study, and we hypothesize that the shared environmental influences might be higher than those we observed in relatively old adults.

Heritability may not be constant across ages [39,40]. Our age moderation analyses (S1 Note) show that the heritability of frequency of public nature space visits reduced with age, driven by increased unique environmental influences. The increased unique environmental influence across age could be due to multiple mechanisms. First, there are more environmental factors that affect elderly individuals in deciding the frequency of their nature space visits, such as accessibility to a green space with specific facilities. Second, the same environmental factors may affect young and old individuals, but these environmental factors have stronger influences on older individuals. Third, the environmental influences may change from generation to generation (i.e., people born in different periods have different experiences), but not with age (i.e., development of individuals). Future research could consider long-term repeated measurements of twin individuals to ascertain the mechanisms underlying changes in the genetic/environmental influences on nature experiences.

### Moderation of the level of urbanization on environmental contributions

The results show reduced unique environmental influences on frequency of public nature space visits in highly urbanized areas. This could be due to limited public nature spaces in highly urbanized areas [9], which may limit the opportunities of nature space visits for most urban residents. This suggests that a person’s (genetically predisposed) nature orientation could be more important in explaining individual differences in frequency of nature space visits for urban residents as they may be more inclined to overcome these barriers to visit nature space in urban settings or to travel to rural areas.

The unique environmental influence on frequency of garden visits increased with the increasing level of urbanization. The uneven access to a garden for urban residents (S1 Fig) might explain the increased environmental influences of garden visits in the highly urbanized areas. Post hoc analyses using only twin individuals in which both twins reported owning a garden (999 twin pairs, 87% of 1,153 twin pairs) showed that the increased unique environmental influence on the frequency of garden visits was no longer evident (S2 Fig, S4 and S5 Tables), suggesting that having access to gardens may be a key factor to explain individual variation in garden visits for urban residents.

In the post hoc analyses using only twin individuals in which both twins reported owning a garden, we also observed that the increased genetic influences on the frequency of garden visits for all twins changed to have a decreasing trend (despite not a statistically significant result, S2 Fig). This reversal may suggest that the changes of genetic influences for garden visits across levels of urbanization ought to be interpreted with caution, as reflected in the large CIs of genetic influences at the high levels of urbanization. In the studied population, fewer participants live in highly urbanized environments, and, among those, few own a garden. This may then have limited the power of the moderation analysis on the genetic influences, and future studies could usefully include more urban residents.

### Limitations and future work

There are several limitations in this study. First, twin analyses assume that there is equal environmental similarity between MZ and DZ twins [41], and the violation of the assumption could lead to an inflation in the estimation of heritability. Second, the shared environmental variance in frequency of public nature space visits was estimated to be negative (although the 95% CI ranges from positive to negative). Negative estimates of variances are possible because there is no lower bound constraint in the models. The negative estimate of shared environmental variance may be due to sampling error as we observe negative but nonsignificant variance [42]. It could also imply that an ADE model (including additive genetic, dominance genetic, and unique environmental influences) may fit better than the ACE model [42]. A larger sample size in future studies would be needed to unpack this further. Third, the estimation of the unique environmental factor includes the measurement error; future studies could quantify the measurement error using repeated measures [43].

Fourth, the perception of nature itself could be more consistent between MZ than between DZ twins. Relatedly, level of urbanization that is unlikely to be affected by differences in perception was the least heritable phenotype. This suggests that our heritability estimates on nature orientation and experiences could be confounded by MZ twins having more similar views on what constitutes nature. Future research could ask twins to rate levels of naturalness of different scenes to test this hypothesis. Fifth, we assume that mating is random. If there is assortative mating on a heritable trait, we may underestimate the heritability and overestimate the shared environmental influence. Last, our studied population is biased toward females (89%). Although we have controlled for sex in our analyses, future studies could benefit from a more gender-balanced cohort.

## Conclusions

Increasing amount of evidence has highlighted the important health benefits of nature experiences, making understanding the drivers behind nature experiences paramount. Our study provides the first evidence of genetic influences on an individual’s orientation toward nature and on nature experiences. Our results also provide evidence of a complex interaction between urbanization and the environmental effects in shaping a person’s nature experience. Consideration of genetic influences on nature experience opens a new dimension to the study of human–nature interactions and helps provide a more comprehensive picture of individual variation in nature experiences.

## Methods

### Participants

Participants were recruited from the largest adult twin registry in the United Kingdom (TwinsUK [25]) and currently live in the UK. The zygosity of participants was assessed by the “peas in a pod” questionnaire and confirmed via genotyping or sequencing [25]. A survey was carried out via an online questionnaire. We obtained responses and outward postcodes from 666 pairs of MZ female twins, 350 pairs of DZ female twins, 98 pairs of MZ male twins, 30 pairs of DZ male twins, and 9 DZ opposite sex twin pairs.

### Ethics statement

This study was approved by the National University of Singapore Institutional Review Board (project number: S-19-266).

### Data collection

#### Nature orientation and nature experiences

The survey sought to measure nature orientation and 4 dimensions of nature experience (questionnaire is included in S2 Note). For measurement of nature orientation, we used the nature relatedness experience subscale, NR-Experience [10]. This scale measures a person’s physical familiarity with the natural world and their level of comfort and desire to be in nature [10]. The responses to 6 statements were collected on a 5-point Likert scale (1 = strongly disagree to 5 = strongly agree). The average score for each participant was then calculated. A higher average score reflects a stronger orientation toward nature.

The nature experience measurements included 4 variables: the frequency and duration of public nature space visits and the frequency and duration of garden visits. Participants were asked the frequency of visits to public nature areas in 8 ranks (0 = never, 1 = less than once every 2 to 3 months, 2 = once every 2 to 3 months, 3 = once a month, 4 = 2 to 3 times a month, 5 = once a week, 6 = 2 to 4 times a week, and 7 = 5 to 7 times a week). Participants were also asked how long they usually spend whenever they visit a public nature area, scored in 7 ranks (1 = up to 30 minutes, 2 = >30 minutes to 1 hour, 3 = >1 to 3 hours, 4 = >3 to 5 hours, 5 = >5 to 7 hours, 6 = >7 to 9 hours, and 7 = >9 hours). When participants indicated that they never visit a public nature space, the duration of public nature space visits was coded as 0.

For the nature experience from garden visits, participants were first asked if they have a garden (Yes or No). Participants were then asked how often they usually spend more than 10 minutes in their gardens (0 = never, 1 = less than once a week, 2 = 2 to 4 times a week, and 3 = 5 to 7 times a week) and how long they usually spend in the garden whenever they visit it (1 = up to 30 minutes, 2 = >30 minutes to 1 hour, 3 = >1 to 3 hours, 4 = >3 to 5 hours, 5 = >5 to 7 hours, 6 = >7 to 9 hours, and 7 = >9 hours). When participants indicated that they do not have their own gardens (92% indicated having a garden), the frequency and duration of garden visits were coded as 0 as they did not have nature experience from garden visits.

To prevent seasonal recall bias in the 4 variables, participants were asked to recall both in spring and summer and in autumn and winter separately, and a yearly rank average of frequency and duration of public nature area visits and garden visits was calculated. An individual with a higher average rank has a higher level of nature experience in that dimension.

The distributions of nature orientation and nature experiences are shown in S3 Fig. The effects of sex and age on the nature orientation, nature experiences, and the level of urbanization of the home district (see below) are shown in S6 Table. The phenotypic correlations partitioned by sex and zygosity are shown in S7–S10 Tables. The intraclass correlations were calculated with 1-way ANOVA fixed effects models using the *psych* package [44].

#### Level of urbanization of the residential district

The level of urbanization of each participant’s residential district was estimated at the district level using the outward postcode of the home address (1,184 unique postcodes). A total of 879 out of 1,153 twin pairs live in different districts. A spatial polygon layer of the outward postcodes for the UK was downloaded from EDINA (Code-Point with Polygons product [45]) under the University of Exeter’s license. We projected the layer to the British grid (EPSG: 27700) before calculating the area of each postcode district polygon. The Land Cover Map 2015 was downloaded as a raster from the UK Centre for Ecology and Hydrology [46] under the University of Exeter’s license (https://www.ceh.ac.uk/services/land-cover-map-2015). The raster was projected to the British grid at a resolution of 25 m^2^ using the nearest neighbor method. Pixels were extracted from the polygon of each postcode and summed by land cover type. A pixel was included if its centroid fell within the polygon. The level of urbanization of each postcode district was calculated as the percentage of urban and suburban (together built-up areas and gardens) land cover categories. The distribution of levels of urbanization is shown in S1 and S3 Figs.

### Twin analyses

#### Multivariate analysis using a direct symmetric approach

To decompose phenotypic variance into additive genetic (A), shared environmental (C), and unique environmental (E) variance, we performed a multivariate analysis with a direct symmetric approach (S4 Fig). The direct symmetric approach allows us to estimate the variance and covariance directly without the constraints of the lower bound of 0 (i.e., variance estimates can be negative), which could avoid bias from a Cholesky decomposition model [42]. The twin analysis assumes that MZ twins share 100% genetic similarity, and DZ twins share 50% genetic similarity. Shared environments between twins are assumed to be identical, and unique environmental factors are assumed to be uncorrelated between the twins and contribute to the differences between twins. In the multivariate analysis, we included nature orientation, level of urbanization of the home district, and 4 dimensions of nature experience (totaling 6 traits). We included level of urbanization because people may choose where to live in a rural to urban gradient. We estimated the genetic and environmental correlations between traits [47]. The genetic correlation between traits indicates the extent to which the genetic factors of 2 traits overlap (if the same genetic factor influences both traits, the genetic correlation is 1). The same interpretation applies to the environmental correlations. We controlled for sex and age by taking the residuals of linear regression models (6 models, each trait as a function of sex and age). This model was run using the *OpenMx* package [48] in R 4.0.2 [49].

#### Moderation analyses

To test the effect of the level of urbanization of the home district on the genetic and environmental influences of nature orientation and nature experiences, we built full bivariate moderation models to examine the gene–environment and environment–environment interactions in the presence of gene–environment and environment–environment correlations [26,27]. We ran 5 models for nature orientation and each dimension of nature experiences. In the moderation models (S5 Fig), the phenotypic variance was partitioned into genetic (Au), shared environmental (Cu), and unique environmental (Eu) variances that are unique to the trait (nature orientation or each dimension of nature experiences) and genetic (Ac), shared environmental (Cc), and unique environmental (Ec) variances that are shared between the moderator (level of urbanization) and trait. The shared variances are intended to capture the covariance between moderator and the trait. Moderation can occur on all 6 variances. Statistically significant moderation on the variance that is unique to the trait (Au, Cu, and Eu) indicates evidence of gene–environment or environment–environment interactions [50]. We then plotted the unstandardized variances explained and heritability [27] as a function of the level of urbanization. We conducted the analyses controlling for age and sex on the traits (not level of urbanization because here level of urbanization was considered as an environmental factor). All models were also run using the *OpenMx* package [48].

## Supporting information

S1 Fig Frequency of urbanization level of the home district (urban; 0 = rural areas, 1 = highly urbanized areas) of participants without and with a domestic garden. (DOCX)

S2 Fig The post hoc analyses only using twin individuals in which both twins reported owning a garden. The thick lines are the estimated variances across the level of urbanization (urban; 0 = rural areas, 1 = highly urbanized areas), and the thin lines are the 95% CIs. CI, confidence interval.
(DOCX)

S3 Fig Frequency of nature orientation, level of urbanization, frequency of public nature space visits (nature frequency), duration of public nature space visits (nature duration), frequency of garden visits (garden frequency), and duration of garden visits (garden duration). Higher value in x-axis represents stronger orientation, higher level of urbanization, and more experiences of nature in different dimensions.
(DOCX)

S4 Fig A multivariate model with a direct symmetric approach with additive genetic, shared environmental, and unique environmental influences. Only additive genetic effects are shown here. T1 = nature orientation, T2 = level of urbanization, T3 = frequency of public nature space visits, T4 = duration of public nature space visit, T5 = frequency of garden visits, and T6 = duration of garden visits.
(DOCX)

S5 Fig Moderation models of additive genetic, shared environmental, and unique environmental influences moderated by the level of urbanization (urban). The phenotypic variance is partitioned into genetic (Au), shared environmental (Cu), and unique environmental (Eu) variances that are unique to the trait and genetic (Ac), shared environmental (Cc), and unique environmental (Ec) variances that are shared between the moderator and trait. Moderation effects of urbanization can occur on all 6 variances.
(DOCX)

S1 Table Intraclass correlation (95% CI) in MZ and DZ twin pairs while controlling for sex and age. Urban = urbanization level. Nature frequency = frequency of public nature space visits. Nature duration = duration of public nature space visits. Garden frequency = frequency of domestic garden visits. Garden duration = duration of domestic garden visits. CI, confidence interval; DZ, dizygotic; MZ, monozygotic.
(DOCX)

S2 Table Path coefficients of the urban moderation models with traits controlling for age and sex. The labels of path coefficient are shown in S5 Fig. Nature frequency = frequency of public nature space visits. Nature duration = duration of public nature space visits. Garden frequency = frequency of domestic garden visits. Garden duration = duration of domestic garden visits.
(DOCX)

S3 Table Model comparisions betwen full moderation models and the models that dropped one moderation parameter. minus2LL = −2*Log-likelihood, df = degrees of freedom. AIC = Akaike information criterion. diffLL = difference in minus2LL. a1t, c1t, and e1t are the moderation effects on genetic, shared environmental, and unique environmental influences that are unique for the phenotype. a1mt, c1mt, and e1mt are the moderation effects on genetic, shared environmental, and unique environmental influences that are shared between the moderator and the phenotype.
(DOCX)

S4 Table Path coefficients of the urban moderation models of frequency and duration of domestic garden visits controlling for age and sex. Only using twin individuals in which both twins reported owning a garden. The labels of path coefficient are shown in S5 Fig.
(DOCX)

S5 Table Model comparisions for frequency and duratio of garden visits between full moderation models and the models that dropped one moderation parameter. Only using twin individuals in which both twins reported owning a garden. minus2LL = −2*Log-likelihood, df = degrees of freedom. AIC = Akaike information criterion. diffLL = difference in minus2LL. a1t, c1t, and e1t are the moderation effects on genetic, shared environmental, and unique environmental influences that are unique for the phenotype. a1mt, c1mt, and e1mt are the moderation effects on genetic, shared environmental, and unique environmental influences that are shared between the moderator and the phenotype.
(DOCX)

S6 Table The linear regressions of the effect of sex and age on each response variable. The standardized residuals of the models were used to run a multivariate model and moderation models with traits controlling for sex and age. Nature frequency = frequency of public nature space visits. Nature duration = duration of public nature space visits. Garden frequency = frequency of domestic garden visits. Garden duration = duration of domestic garden visits. Urban = urbanization level.
(DOCX)

S7 Table Between-twin within and across trait correlations of MZ females (Pearson correlation). Urban = urbanization level. Nature duration = duration of public nature space visits. Nature frequency = frequency of public nature space visits. Garden duration = duration of domestic garden visits. Garden frequency = frequency of domestic garden visits. MZ, monozygotic.
(DOCX)

S8 Table Between-twin within and across trait correlations of DZ females (Pearson correlation). Urban = urbanization level. Nature duration = duration of public nature space visits. Nature frequency = frequency of public nature space visits. Garden duration = duration of domestic garden visits. Garden frequency = frequency of domestic garden visits. DZ, dizygotic.
(DOCX)

S9 Table Between-twin within and across trait correlations of MZ males (Pearson correlation). Urban = urbanization level. Nature duration = duration of public nature space visits. Nature frequency = frequency of public nature space visits. Garden duration = duration of domestic garden visits. Garden frequency = frequency of domestic garden visits. MZ, monozygotic.
(DOCX)

S10 Table Between-twin within and across trait correlations of DZ males (Pearson correlation). Urban = urbanization level. Nature duration = duration of public nature space visits. Nature frequency = frequency of public nature space visits. Garden duration = duration of domestic garden visits. Garden frequency = frequency of domestic garden visits. DZ, dizygotic.
(DOCX)

S1 Note Age moderation analyses. (DOCX)

S2 Note Questions and statements used to measure nature orientation and nature experiences. (DOCX)

## Abbreviations

CI: confidence interval
DZ: dizygotic
MZ: monozygotic
