# Supplementary material for: People’s desire to be in nature and how they experience it are partially heritable
Source: PLoS Biol. 2022 Feb 3;20(2):e3001500. doi: 10.1371/journal.pbio.3001500 (PMC8812842; doi:10.1371/journal.pbio.3001500)
Supplement: S1 Fig — (DOCX) [file pbio.3001500.s001.docx]

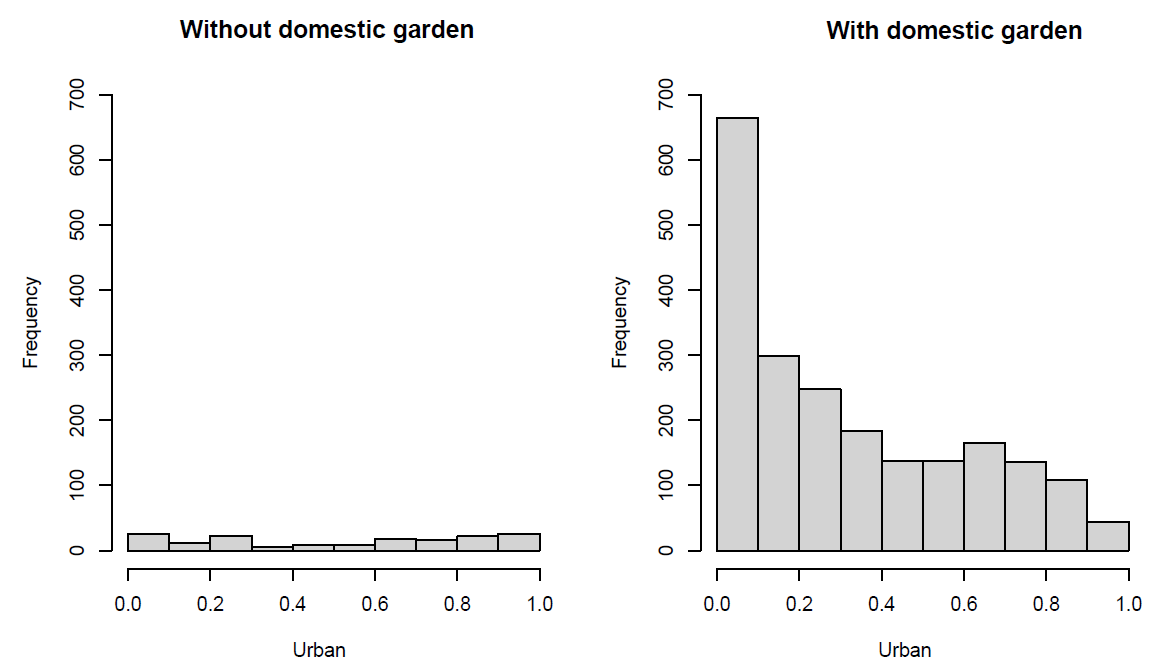


S1 Fig. Frequency of urbanization level of the home district (urban; 0 = rural areas, 1 = highly urbanized areas) of participants without and with a domestic garden.
