# Supplementary material for: People’s desire to be in nature and how they experience it are partially heritable
Source: PLoS Biol. 2022 Feb 3;20(2):e3001500. doi: 10.1371/journal.pbio.3001500 (PMC8812842; doi:10.1371/journal.pbio.3001500)
Supplement: S2 Fig — The thick lines are the estimated variances across the level of urbanization (urban; 0 = rural areas, 1 = highly urbanized areas), and the thin lines are the 95% CIs. CI, confidence interval. (DOCX) [file pbio.3001500.s002.docx]

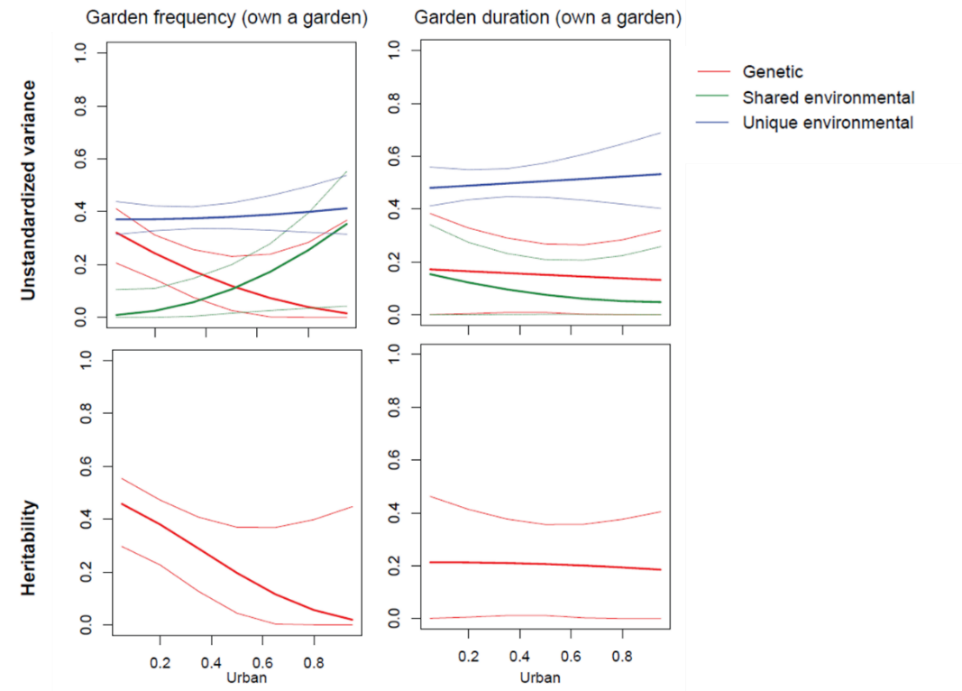


S2 Fig. The post-hoc analyses only using twin individuals in which both twins reported owning a garden. The thick lines are the estimated variances across the level of urbanization (urban; 0 = rural areas, 1 = highly urbanized areas), and the thin lines are the 95% CIs.
