# Supplementary material for: People’s desire to be in nature and how they experience it are partially heritable
Source: PLoS Biol. 2022 Feb 3;20(2):e3001500. doi: 10.1371/journal.pbio.3001500 (PMC8812842; doi:10.1371/journal.pbio.3001500)
Supplement: S3 Fig — Higher value in x-axis represents stronger orientation, higher level of urbanization, and more experiences of nature in different dimensions. (DOCX) [file pbio.3001500.s003.docx]

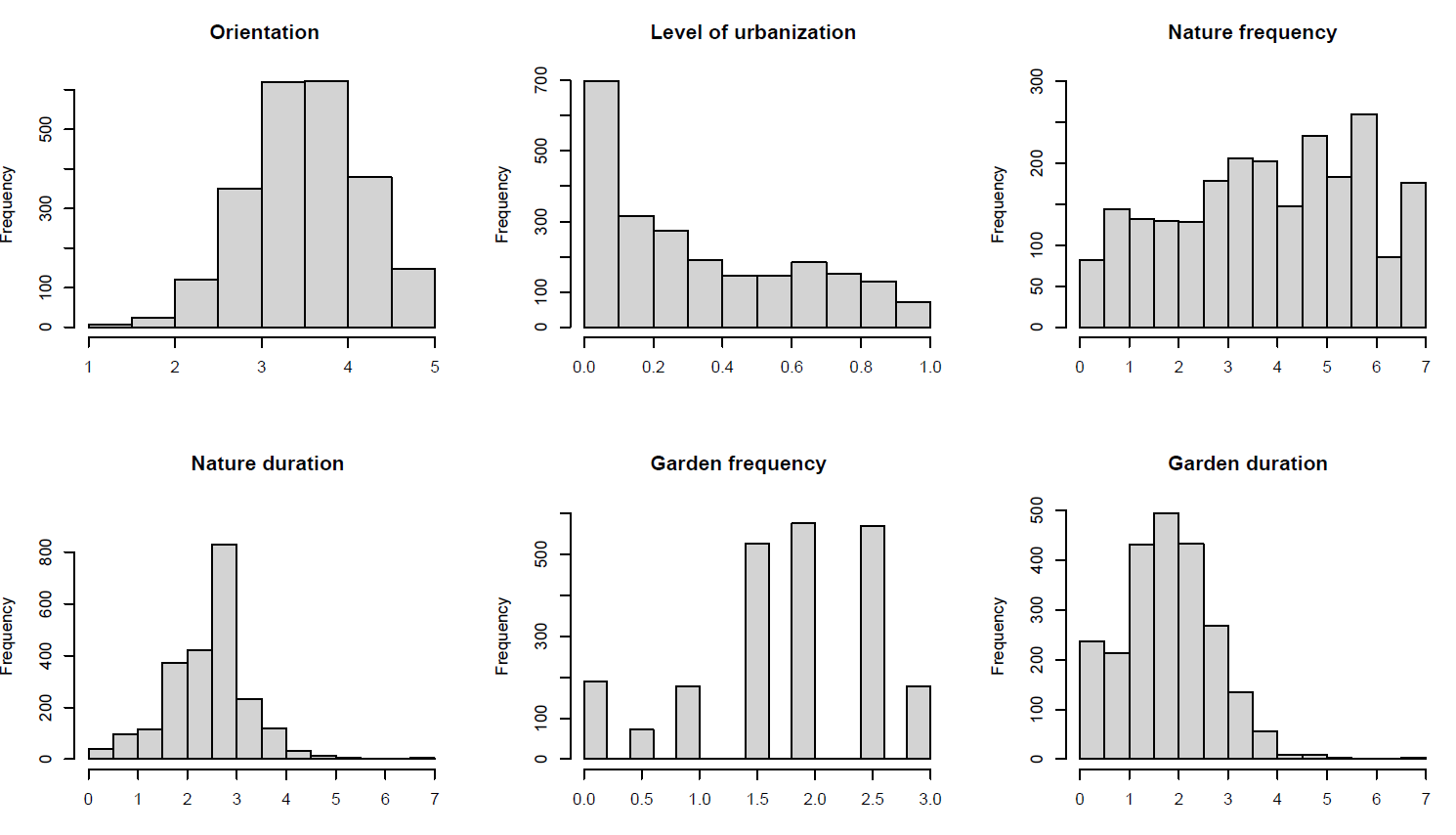


S3 Fig. Frequency of nature orientation, level of urbanization, frequency of public nature space visits (Nature frequency), duration of public nature space visits (Nature duration), frequency of garden visits (Garden frequency), and duration of garden visits (Garden duration). Higher value in x-axis represents stronger orientation, higher level of urbanization, and more experiences of nature in different dimensions.
