# Supplementary material for: People’s desire to be in nature and how they experience it are partially heritable
Source: PLoS Biol. 2022 Feb 3;20(2):e3001500. doi: 10.1371/journal.pbio.3001500 (PMC8812842; doi:10.1371/journal.pbio.3001500)
Supplement: S4 Fig — Only additive genetic effects are shown here. T1 = nature orientation, T2 = level of urbanization, T3 = frequency of public nature space visits, T4 = duration of public nature space visit, T5 = frequency of garden visits, and T6 = duration of garden visits. (DOCX) [file pbio.3001500.s004.docx]

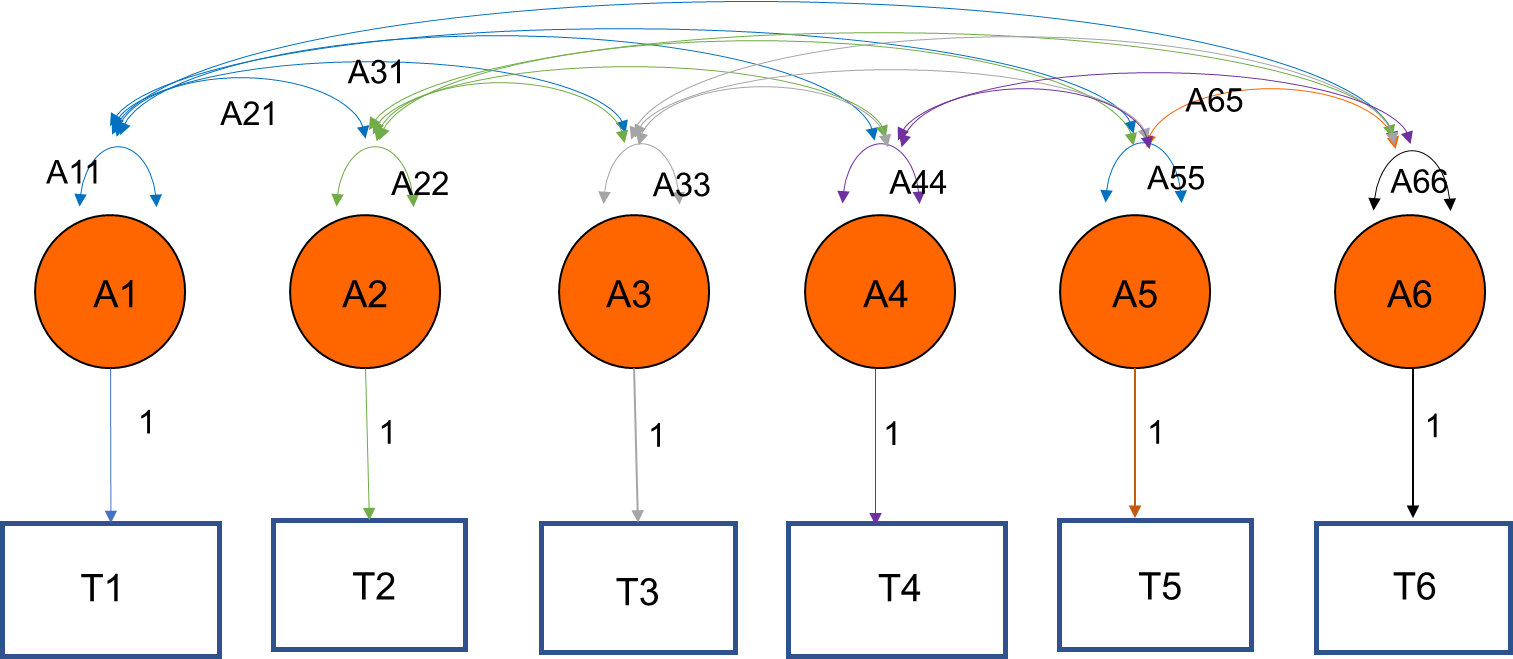


S4 Fig. A multivariate model with a direct symmetric approach with additive genetic effects, shared environmental, and unique environmental influences. Only additive effects are shown here. T1 = nature orientation, T2 = level of urbanization, T3 = frequency of public nature space visits, T4 = duration of public nature space visit, T5 = frequency of garden visits, T6 = duration of garden visits.
