# Supplementary material for: People’s desire to be in nature and how they experience it are partially heritable
Source: PLoS Biol. 2022 Feb 3;20(2):e3001500. doi: 10.1371/journal.pbio.3001500 (PMC8812842; doi:10.1371/journal.pbio.3001500)
Supplement: S5 Fig — The phenotypic variance is partitioned into genetic (Au), shared environmental (Cu), and unique environmental (Eu) variances that are unique to the trait and genetic (Ac), shared environmental (Cc), and unique environmental (Ec) variances that are shared between the moderator and trait. Moderation effects of urbanization can occur on all 6 variances. (DOCX) [file pbio.3001500.s005.docx]

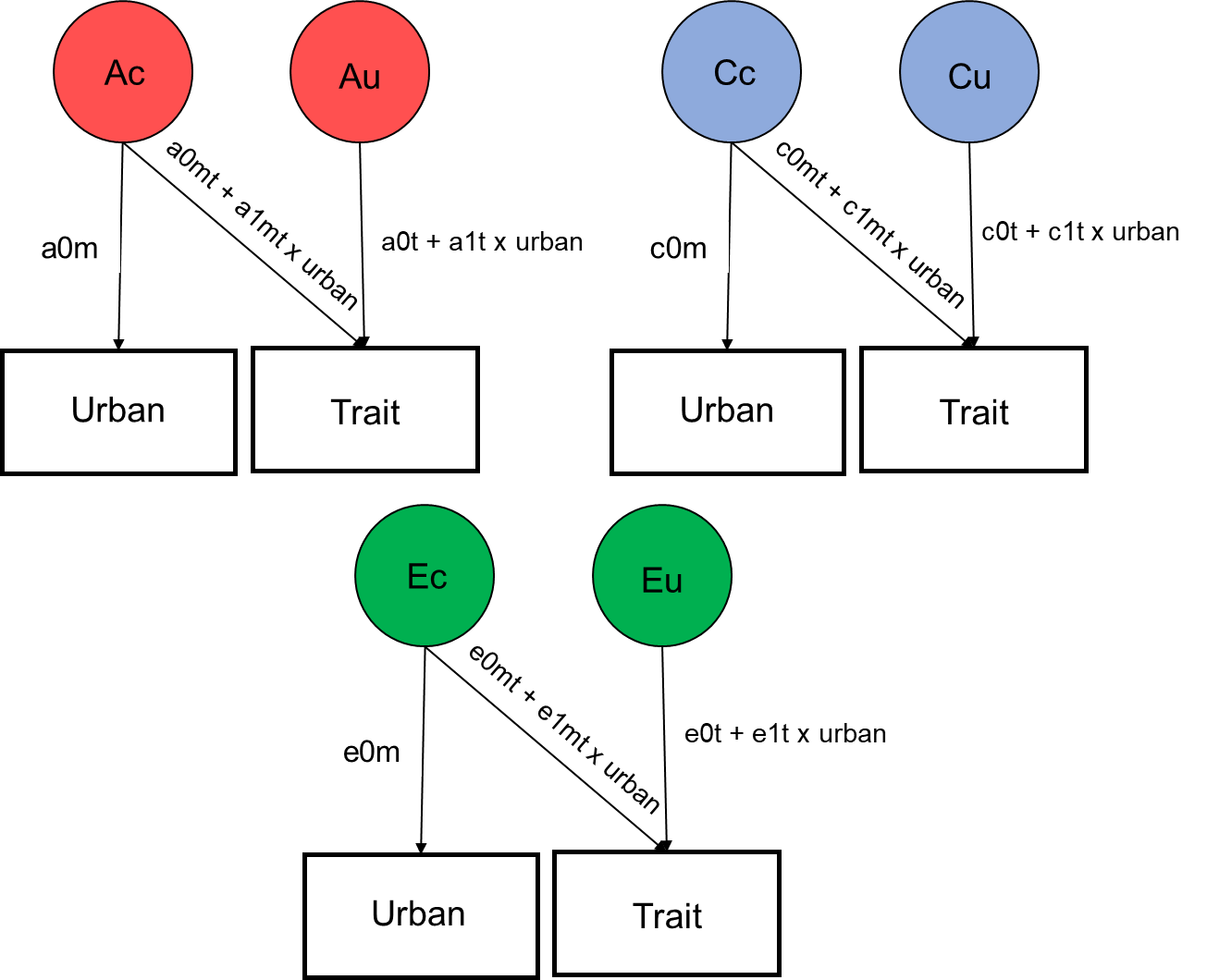


S5 Fig. Moderation models of additive genetic, shared environmental, and unique environmental influences moderated by the level of urbanization (urban). The phenotypic variance is partitioned into genetic (Au), shared environmental (Cu), and unique environmental (Eu) variances that are unique to the trait and genetic (Ac), shared environmental (Cc), and unique environmental (Ec) variances that are shared between the moderator and trait. Moderation effects of urbanization can occur on all six variances.
