# Supplementary material for: People’s desire to be in nature and how they experience it are partially heritable
Source: PLoS Biol. 2022 Feb 3;20(2):e3001500. doi: 10.1371/journal.pbio.3001500 (PMC8812842; doi:10.1371/journal.pbio.3001500)
Supplement: S1 Table — Urban = urbanization level. Nature frequency = frequency of public nature space visits. Nature duration = duration of public nature space visits. Garden frequency = frequency of domestic garden visits. Garden duration = duration of domestic garden visits. CI, confidence interval; DZ, dizygotic; MZ, monozygotic. (DOCX) [file pbio.3001500.s006.docx]

S1 Table. Intraclass correlation (95% CI) in monozygotic and dizygotic twin pairs while controlling for sex and age. Urban = urbanization level. Nature frequency = frequency of public nature space visits. Nature duration = duration of public nature space visits. Garden frequency = frequency of domestic garden visits. Garden duration = duration of domestic garden visits.

|  | Monozygotic | Dizygotic |
| --- | --- | --- |
| Urban | 0.42 (0.37 - 0.47) | 0.39 (0.32 - 0.46) |
| Orientation | 0.49 (0.45 - 0.54) | 0.26 (0.19 - 0.34) |
| Nature frequency | 0.38 (0.33 - 0.43) | 0.16 (0.08 - 0.24) |
| Nature duration | 0.25 (0.19 - 0.30) | 0.15 (0.07 - 0.23) |
| Garden frequency | 0.39 (0.34 - 0.44) | 0.20 (0.12 - 0.28) |
| Garden duration | 0.37 (0.31 - 0.42) | 0.16 (0.08 - 0.24) |
