# Supplementary material for: People’s desire to be in nature and how they experience it are partially heritable
Source: PLoS Biol. 2022 Feb 3;20(2):e3001500. doi: 10.1371/journal.pbio.3001500 (PMC8812842; doi:10.1371/journal.pbio.3001500)
Supplement: S2 Table — The labels of path coefficient are shown in S5 Fig. Nature frequency = frequency of public nature space visits. Nature duration = duration of public nature space visits. Garden frequency = frequency of domestic garden visits. Garden duration = duration of domestic garden visits. (DOCX) [file pbio.3001500.s007.docx]

S2 Table. Path coefficients of the urban moderation models with traits controlling for age and sex. The labels of path coefficient are shown in S5 Fig. Nature frequency = frequency of public nature space visits. Nature duration = duration of public nature space visits. Garden frequency = frequency of domestic garden visits. Garden duration = duration of domestic garden visits.

|  | Orientation | | Nature frequency | | Nature duration | | Garden frequency | | Garden duration | |
| --- | --- | --- | --- | --- | --- | --- | --- | --- | --- | --- |
|  | Estimate | SE | Estimate | SE | Estimate | SE | Estimate | SE | Estimate | SE |
| a0m | 0.07 | 0.06 | 0.07 | 0.05 | 0.08 | 0.04 | 0.07 | 0.05 | 0.06 | 0.06 |
| c0m | 0.17 | 0.02 | 0.17 | 0.02 | 0.17 | 0.02 | 0.17 | 0.02 | 0.18 | 0.02 |
| e0m | 0.21 | 0.01 | 0.21 | 0.01 | 0.21 | <0.01 | 0.21 | 0.01 | 0.21 | 0.01 |
| a0t | 0.60 | 0.15 | 0.54 | 0.13 | -0.58 | 0.19 | 0.40 | 0.29 | 0.35 | 0.15 |
| c0t | 0.17 | 0.32 | <0.01 | 0.31 | <0.01 | 1.76 | -0.39 | 0.22 | -0.55 | 0.10 |
| e0t | 0.72 | 0.03 | 0.83 | 0.03 | 0.80 | 0.03 | 0.70 | 0.03 | 0.75 | 0.03 |
| a1t | 0.12 | 0.24 | 0.13 | 0.21 | 0.83 | 0.26 | 0.32 | 0.46 | 0.30 | 0.23 |
| c1t | 0.04 | 0.60 | <0.01 | 0.55 | <0.01 | 0.73 | 0.72 | 0.52 | 0.71 | 0.17 |
| e1t | -0.04 | 0.07 | -0.17 | 0.08 | 0.12 | 0.08 | 0.17 | 0.08 | 0.11 | 0.08 |
| a0mt | -0.27 | 0.31 | 0.26 | 0.29 | 0.23 | 0.36 | 0.33 | 0.37 | 0.02 | 0.31 |
| c0mt | -0.12 | 0.12 | 0.03 | 0.12 | -0.11 | 0.15 | -0.14 | 0.15 | -0.06 | 0.12 |
| e0mt | -0.27 | 0.06 | -0.10 | 0.06 | -0.08 | 0.06 | -0.10 | 0.06 | -0.01 | 0.06 |
| a1mt | 0.32 | 0.35 | -0.44 | 0.36 | 0.20 | 0.52 | -0.51 | 0.69 | -0.17 | 0.44 |
| c1mt | -0.09 | 0.18 | -0.28 | 0.20 | -0.26 | 0.24 | -0.15 | 0.29 | -0.15 | 0.18 |
| e1mt | 0.41 | 0.12 | 0.36 | 0.11 | 0.08 | 0.12 | 0.08 | 0.12 | -0.07 | 0.12 |
