# Supplementary material for: People’s desire to be in nature and how they experience it are partially heritable
Source: PLoS Biol. 2022 Feb 3;20(2):e3001500. doi: 10.1371/journal.pbio.3001500 (PMC8812842; doi:10.1371/journal.pbio.3001500)
Supplement: S3 Table — minus2LL = −2*Log-likelihood, df = degrees of freedom. AIC = Akaike information criterion. diffLL = difference in minus2LL. a1t, c1t, and e1t are the moderation effects on genetic, shared environmental, and unique environmental influences that are unique for the phenotype. a1mt, c1mt, and e1mt are the moderation effects on genetic, shared environmental, and unique environmental influences that are shared between the moderator and the phenotype. (DOCX) [file pbio.3001500.s008.docx]

S3 Table. Model comparisions betwen full moderation models and the models that dropped one moderation parameter. minus2LL = -2*Log-likelihood, df = degrees of freedom. AIC = Akaike information criterion. diffLL = difference in minus2LL. a1t, c1t, e1t are the moderation effects on genetic, shared environmental and unique environmental influences that are unique for the phenotype. a1mt, c1mt, e1mt are the moderation effects on genetic, shared environmental and unique environmental influences that are shared between the moderator and the phenotype.

| Nature orientation | |  |  |  |  |  |
| --- | --- | --- | --- | --- | --- | --- |
|  | Model | minus2LL | df | AIC | diffLL | p |
| 1 | Full moderation | 6651.91 | 4557 | -2462.09 | NA | NA |
| 2 | Drop a1t | 6652.14 | 4558 | -2463.86 | 0.23 | 0.631 |
| 3 | Drop c1t | 6651.92 | 4558 | -2464.09 | 0.00 | 0.950 |
| 4 | Drop e1t | 6652.35 | 4558 | -2463.65 | 0.44 | 0.508 |
| 5 | Drop a1mt | 6652.24 | 4558 | -2463.76 | 0.32 | 0.569 |
| 6 | Drop c1mt | 6652.18 | 4558 | -2463.82 | 0.27 | 0.604 |
| 7 | Drop e1mt | 6664.73 | 4558 | -2451.27 | 12.82 | <0.001 |
| Nature frequency | |  |  |  |  |  |
|  | Model | minus2LL | df | AIC | diffLL | p |
| 1 | Full moderation | 6872.67 | 4579 | -2285.33 | NA | NA |
| 2 | Drop a1t | 6873.09 | 4580 | -2286.91 | 0.42 | 0.518 |
| 3 | Drop c1t | 6872.67 | 4580 | -2287.33 | 0.00 | 1.000 |
| 4 | Drop e1t | 6876.92 | 4580 | -2283.08 | 4.24 | 0.039 |
| 5 | Drop a1mt | 6873.20 | 4580 | -2286.80 | 0.53 | 0.468 |
| 6 | Drop c1mt | 6874.63 | 4580 | -2285.37 | 1.96 | 0.162 |
| 7 | Drop e1mt | 6881.56 | 4580 | -2278.44 | 8.89 | 0.003 |
| Nature duration | |  |  |  |  |  |
|  | Model | minus2LL | df | AIC | diffLL | p |
| 1 | Full moderation | 6939.25 | 4579 | -2218.76 | NA | NA |
| 2 | Drop a1t | 6940.87 | 4580 | -2219.14 | 1.62 | 0.203 |
| 3 | Drop c1t | 6939.25 | 4580 | -2220.76 | 0.00 | 1.000 |
| 4 | Drop e1t | 6941.49 | 4580 | -2218.51 | 2.25 | 0.134 |
| 5 | Drop a1mt | 6939.36 | 4580 | -2220.64 | 0.12 | 0.729 |
| 6 | Drop c1mt | 6940.19 | 4580 | -2219.81 | 0.95 | 0.330 |
| 7 | Drop e1mt | 6939.75 | 4580 | -2220.25 | 0.51 | 0.477 |
| Garden frequency | |  |  |  |  |  |
|  | Model | minus2LL | df | AIC | diffLL | p |
| 1 | Full moderation | 6792.33 | 4577 | -2361.67 | NA | NA |
| 2 | Drop a1t | 6792.81 | 4578 | -2363.19 | 0.48 | 0.490 |
| 3 | Drop c1t | 6792.99 | 4578 | -2363.01 | 0.66 | 0.418 |
| 4 | Drop e1t | 6796.94 | 4578 | -2359.06 | 4.60 | 0.032 |
| 5 | Drop a1mt | 6792.73 | 4578 | -2363.28 | 0.39 | 0.532 |
| 6 | Drop c1mt | 6792.61 | 4578 | -2363.39 | 0.28 | 0.598 |
| 7 | Drop e1mt | 6792.74 | 4578 | -2363.26 | 0.41 | 0.524 |
| Garden duration | |  |  |  |  |  |
|  | Model | minus2LL | df | AIC | diffLL | p |
| 1 | Full moderation | 6845.76 | 4577 | -2308.24 | NA | NA |
| 2 | Drop a1t | 6846.01 | 4578 | -2309.99 | 0.25 | 0.618 |
| 3 | Drop c1t | 6850.22 | 4578 | -2305.78 | 4.46 | 0.035 |
| 4 | Drop e1t | 6847.67 | 4578 | -2308.33 | 1.91 | 0.167 |
| 5 | Drop a1mt | 6845.90 | 4578 | -2310.11 | 0.14 | 0.713 |
| 6 | Drop c1mt | 6846.31 | 4578 | -2309.70 | 0.55 | 0.460 |
| 7 | Drop e1mt | 6846.11 | 4578 | -2309.89 | 0.35 | 0.556 |
