# Supplementary material for: People’s desire to be in nature and how they experience it are partially heritable
Source: PLoS Biol. 2022 Feb 3;20(2):e3001500. doi: 10.1371/journal.pbio.3001500 (PMC8812842; doi:10.1371/journal.pbio.3001500)
Supplement: S4 Table — Only using twin individuals in which both twins reported owning a garden. The labels of path coefficient are shown in S5 Fig. (DOCX) [file pbio.3001500.s009.docx]

S4 Table. Path coefficients of the urban moderation models of frequency and duration of domestic garden visits controlling for age and sex. Only using twin individuals in which both twins reported owning a garden. The labels of path coefficient are shown in S5 Fig.

|  | Garden frequency | | Garden duration | |
| --- | --- | --- | --- | --- |
|  | Estimate | SE | Estimate | SE |
| a0m | 0.04 | 0.03 | 0.04 | 0.04 |
| c0m | 0.17 | 0.01 | 0.17 | 0.01 |
| e0m | 0.21 | <0.01 | 0.21 | <0.01 |
| a0t | <0.01 | 4.39 | <0.01 | 0.92 |
| c0t | -0.02 | 0.15 | 0.38 | 0.17 |
| e0t | 0.60 | 0.03 | 0.69 | 0.03 |
| a1t | <0.01 | 4.60 | <0.01 | 0.49 |
| c1t | 0.58 | 0.18 | -0.34 | 0.28 |
| e1t | 0.04 | 0.06 | 0.04 | 0.07 |
| a0mt | 0.59 | 0.05 | 0.42 | 0.15 |
| c0mt | -0.08 | 0.10 | -0.14 | 0.11 |
| e0mt | -0.13 | 0.05 | -0.02 | 0.05 |
| a1mt | -0.49 | 0.19 | -0.06 | 0.22 |
| c1mt | -0.18 | 0.13 | -0.08 | 0.12 |
| e1mt | 0.23 | 0.10 | 0.03 | 0.11 |
