# Supplementary material for: People’s desire to be in nature and how they experience it are partially heritable
Source: PLoS Biol. 2022 Feb 3;20(2):e3001500. doi: 10.1371/journal.pbio.3001500 (PMC8812842; doi:10.1371/journal.pbio.3001500)
Supplement: S6 Table — The standardized residuals of the models were used to run a multivariate model and moderation models with traits controlling for sex and age. Nature frequency = frequency of public nature space visits. Nature duration = duration of public nature space visits. Garden frequency = frequency of domestic garden visits. Garden duration = duration of domestic garden visits. Urban = urbanization level. (DOCX) [file pbio.3001500.s011.docx]

S6 Table. The linear regressions of the effect of sex and age on each response variable. The standardized residuals of the models were used to run a multivariate model and moderation models with traits controlling for sex and age. Nature frequency = frequency of public nature space visits. Nature duration = duration of public nature space visits. Garden frequency = frequency of domestic garden visits. Garden duration = duration of domestic garden visits. Urban = urbanization level.

| Response variable |  | Estimate | SE | t value | Pr(>\|t\|) |
| --- | --- | --- | --- | --- | --- |
| Orientation | (Intercept) | 3.732 | 0.060 | 62.595 | < 0.001 |
|  | Sex (male) | 0.146 | 0.043 | 3.382 | <0.001 |
|  | Age | -0.003 | 0.001 | -2.873 | 0.004 |
| Nature frequency | (Intercept) | 4.796 | 0.171 | 28.089 | < 0.001 |
|  | Sex (male) | -0.094 | 0.124 | -0.758 | 0.449 |
|  | Age | -0.013 | 0.003 | -4.627 | < 0.001 |
| Nature duration | (Intercept) | 2.785 | 0.078 | 35.779 | < 0.001 |
|  | Sex (male) | 0.110 | 0.056 | 1.955 | 0.051 |
|  | Age | -0.002 | 0.001 | -1.493 | 0.136 |
| Garden frequency | (Intercept) | 0.647 | 0.068 | 9.473 | < 0.001 |
|  | Sex (male) | -0.054 | 0.049 | -1.098 | 0.272 |
|  | Age | 0.019 | 0.001 | 17.424 | < 0.001 |
| Garden duration | (Intercept) | 0.733 | 0.088 | 8.369 | < 0.001 |
|  | Sex (male) | -0.038 | 0.063 | -0.605 | 0.545 |
|  | Age | 0.021 | 0.001 | 14.876 | < 0.001 |
| Urban | (Intercept) | 0.530 | 0.025 | 20.780 | < 0.001 |
|  | Sex (male) | -0.0005 | 0.018 | -0.027 | 0.978 |
|  | Age | -0.003 | <0.001 | -7.791 | < 0.001 |
