# Supplementary material for: People’s desire to be in nature and how they experience it are partially heritable
Source: PLoS Biol. 2022 Feb 3;20(2):e3001500. doi: 10.1371/journal.pbio.3001500 (PMC8812842; doi:10.1371/journal.pbio.3001500)
Supplement: S7 Table — Urban = urbanization level. Nature duration = duration of public nature space visits. Nature frequency = frequency of public nature space visits. Garden duration = duration of domestic garden visits. Garden frequency = frequency of domestic garden visits. MZ, monozygotic. (DOCX) [file pbio.3001500.s012.docx]

S7 Table. Between-twin within and across trait correlations of monozygotic females (Pearson correlation). Urban = urbanization level. Nature duration = duration of public nature space visits. Nature frequency = frequency of public nature space visits. Garden duration = duration of domestic garden visits. Garden frequency = frequency of domestic garden visits.

| R | Urban | Orientation | Nature duration | Nature frequency | Garden duration | Garden frequency |
| --- | --- | --- | --- | --- | --- | --- |
| Urban | 0.44 | -0.12 | -0.05 | -0.01 | -0.17 | -0.15 |
| Orientation | -0.07 | 0.5 | 0.15 | 0.21 | 0.16 | 0.15 |
| Nature duration | -0.05 | 0.16 | 0.27 | 0.1 | 0.09 | 0.05 |
| Nature frequency | <0.01 | 0.3 | 0.12 | 0.4 | -0.01 | 0.08 |
| Garden duration | -0.15 | 0.17 | 0.11 | 0.01 | 0.45 | 0.37 |
| Garden frequency | -0.21 | 0.2 | 0.09 | 0.11 | 0.43 | 0.47 |
| P value |  |  |  |  |  |  |
| Urban | <0.001 | 0.002 | 0.177 | 0.881 | <0.001 | <0.001 |
| Orientation | 0.085 | <0.001 | <0.001 | <0.001 | <0.001 | <0.001 |
| Nature duration | 0.191 | <0.001 | <0.001 | 0.009 | 0.016 | 0.247 |
| Nature frequency | 0.919 | <0.001 | 0.002 | <0.001 | 0.730 | 0.047 |
| Garden duration | <0.001 | <0.001 | 0.005 | 0.824 | <0.001 | <0.001 |
| Garden frequency | <0.001 | <0.001 | 0.016 | 0.006 | <0.001 | <0.001 |
