# Supplementary material for: People’s desire to be in nature and how they experience it are partially heritable
Source: PLoS Biol. 2022 Feb 3;20(2):e3001500. doi: 10.1371/journal.pbio.3001500 (PMC8812842; doi:10.1371/journal.pbio.3001500)
Supplement: S8 Table — Urban = urbanization level. Nature duration = duration of public nature space visits. Nature frequency = frequency of public nature space visits. Garden duration = duration of domestic garden visits. Garden frequency = frequency of domestic garden visits. DZ, dizygotic. (DOCX) [file pbio.3001500.s013.docx]

S8 Table. Between-twin within and across trait correlations of dizygotic females (Pearson correlation). Urban = urbanization level. Nature duration = duration of public nature space visits. Nature frequency = frequency of public nature space visits. Garden duration = duration of domestic garden visits. Garden frequency = frequency of domestic garden visits.

| R | Urban | Orientation | Nature duration | Nature frequency | Garden duration | Garden frequency |
| --- | --- | --- | --- | --- | --- | --- |
| Urban | 0.4 | -0.07 | -0.09 | -0.19 | -0.08 | -0.14 |
| Orientation | -0.15 | 0.27 | 0.13 | 0.18 | 0.22 | 0.16 |
| Nature duration | -0.07 | <0.01 | 0.15 | 0.04 | 0.06 | 0.03 |
| Nature frequency | 0.05 | 0.1 | 0.02 | 0.15 | 0.03 | 0.04 |
| Garden duration | -0.12 | 0.04 | 0.01 | -0.01 | 0.22 | 0.18 |
| Garden frequency | -0.11 | 0.08 | -0.01 | 0.01 | 0.26 | 0.27 |
| P value |  |  |  |  |  |  |
| Urban | <0.001 | 0.181 | 0.085 | <0.001 | 0.158 | 0.011 |
| Orientation | 0.005 | <0.001 | 0.013 | 0.001 | <0.001 | 0.003 |
| Nature duration | 0.199 | 0.940 | 0.004 | 0.439 | 0.291 | 0.564 |
| Nature frequency | 0.309 | 0.067 | 0.708 | 0.004 | 0.536 | 0.421 |
| Garden duration | 0.023 | 0.465 | 0.825 | 0.908 | <0.001 | 0.001 |
| Garden frequency | 0.048 | 0.130 | 0.804 | 0.851 | <0.001 | <0.001 |
