# Supplementary material for: People’s desire to be in nature and how they experience it are partially heritable
Source: PLoS Biol. 2022 Feb 3;20(2):e3001500. doi: 10.1371/journal.pbio.3001500 (PMC8812842; doi:10.1371/journal.pbio.3001500)
Supplement: S9 Table — Urban = urbanization level. Nature duration = duration of public nature space visits. Nature frequency = frequency of public nature space visits. Garden duration = duration of domestic garden visits. Garden frequency = frequency of domestic garden visits. MZ, monozygotic. (DOCX) [file pbio.3001500.s014.docx]

S9 Table. Between-twin within and across trait correlations of monozygotic males (Pearson correlation). Urban = urbanization level. Nature duration = duration of public nature space visits. Nature frequency = frequency of public nature space visits. Garden duration = duration of domestic garden visits. Garden frequency = frequency of domestic garden visits.

| R | Urban | Orientation | Nature duration | Nature frequency | Garden duration | Garden frequency |
| --- | --- | --- | --- | --- | --- | --- |
| Urban | 0.39 | 0.02 | 0.03 | <0.01 | -0.27 | -0.18 |
| Orientation | 0.01 | 0.48 | 0.1 | 0.33 | 0.16 | 0.27 |
| Nature duration | -0.01 | 0.21 | 0.12 | 0.25 | 0.15 | 0.2 |
| Nature frequency | -0.04 | 0.19 | 0.17 | 0.29 | -0.04 | 0.01 |
| Garden duration | -0.08 | 0.18 | 0.13 | 0.19 | 0.38 | 0.32 |
| Garden frequency | -0.1 | 0.31 | 0.13 | 0.26 | 0.42 | 0.49 |
| P value |  |  |  |  |  |  |
| Urban | <0.001 | 0.858 | 0.801 | 0.988 | 0.008 | 0.080 |
| Orientation | 0.936 | <0.001 | 0.314 | 0.001 | 0.115 | 0.008 |
| Nature duration | 0.902 | 0.039 | 0.233 | 0.016 | 0.154 | 0.056 |
| Nature frequency | 0.669 | 0.060 | 0.093 | 0.004 | 0.726 | 0.895 |
| Garden duration | 0.449 | 0.076 | 0.217 | 0.061 | <0.001 | 0.001 |
| Garden frequency | 0.339 | 0.002 | 0.196 | 0.012 | <0.001 | <0.001 |
