# Supplementary material for: People’s desire to be in nature and how they experience it are partially heritable
Source: PLoS Biol. 2022 Feb 3;20(2):e3001500. doi: 10.1371/journal.pbio.3001500 (PMC8812842; doi:10.1371/journal.pbio.3001500)
Supplement: S10 Table — Urban = urbanization level. Nature duration = duration of public nature space visits. Nature frequency = frequency of public nature space visits. Garden duration = duration of domestic garden visits. Garden frequency = frequency of domestic garden visits. DZ, dizygotic. (DOCX) [file pbio.3001500.s015.docx]

S10 Table. Between-twin within and across trait correlations of dizygotic males (Pearson correlation). Urban = urbanization level. Nature duration = duration of public nature space visits. Nature frequency = frequency of public nature space visits. Garden duration = duration of domestic garden visits. Garden frequency = frequency of domestic garden visits.

| R | Urban | Orientation | Nature duration | Nature frequency | Garden duration | Garden frequency |
| --- | --- | --- | --- | --- | --- | --- |
| Urban | 0.31 | 0.09 | -0.13 | 0.02 | 0.25 | -0.03 |
| Orientation | 0.07 | 0.45 | 0.5 | 0.3 | -0.21 | -0.19 |
| Nature duration | -0.23 | 0.06 | 0.31 | 0.37 | 0.06 | 0.24 |
| Nature frequency | <0.01 | <0.01 | 0.21 | 0.36 | -0.12 | -0.04 |
| Garden duration | -0.23 | -0.01 | 0.07 | 0.15 | -0.09 | 0.04 |
| Garden frequency | -0.26 | 0.12 | 0.26 | 0.38 | 0.02 | 0.04 |
| P value |  |  |  |  |  |  |
| Urban | 0.094 | 0.627 | 0.499 | 0.923 | 0.186 | 0.875 |
| Orientation | 0.722 | 0.015 | 0.006 | 0.116 | 0.263 | 0.304 |
| Nature duration | 0.229 | 0.759 | 0.100 | 0.046 | 0.763 | 0.198 |
| Nature frequency | 0.985 | 0.985 | 0.266 | 0.054 | 0.513 | 0.824 |
| Garden duration | 0.231 | 0.957 | 0.710 | 0.449 | 0.628 | 0.844 |
| Garden frequency | 0.170 | 0.524 | 0.175 | 0.043 | 0.919 | 0.834 |
