# Supplementary material for: People’s desire to be in nature and how they experience it are partially heritable
Source: PLoS Biol. 2022 Feb 3;20(2):e3001500. doi: 10.1371/journal.pbio.3001500 (PMC8812842; doi:10.1371/journal.pbio.3001500)
Supplement: S1 Note — (DOCX) [file pbio.3001500.s016.docx]

S1 Note. Age moderation analyses

The age distribution is shown in Fig A. To examine the change of genetic and environmental influences with age, we ran moderation models (ACE-XYZ-M model in [1]) for each phenotype (nature orientation and four dimensions of nature experience). In the models (Fig B), the phenotypic variance is partitioned into genetic (A), shared environmental (C), and unique environmental (E) variances. The moderation of age can occur on all three variances, and the moderator also can influence the mean (main effect) as the phenotypic regression. We performed the moderation analyses with controlling for sex. The changes of genetic and environmental influences with age are shown in Fig C. The path coefficients are shown in Table A. The model comparisons are shown in Table B.

For nature orientation, the genetic influence reduced with age, but the moderation effect was not statistically significant. For frequency and duration of public nature space visits, the unique environmental influences significantly increased with age. For frequency and duration of garden visits, the models showed U-shaped changes in the shared environmental influences with age but the lower bound of CIs were close to zero.

Reference:

1. Purcell S. Variance components models for gene-environment interaction in twin analysis. Twin Res. 2002;5(6):554-71.


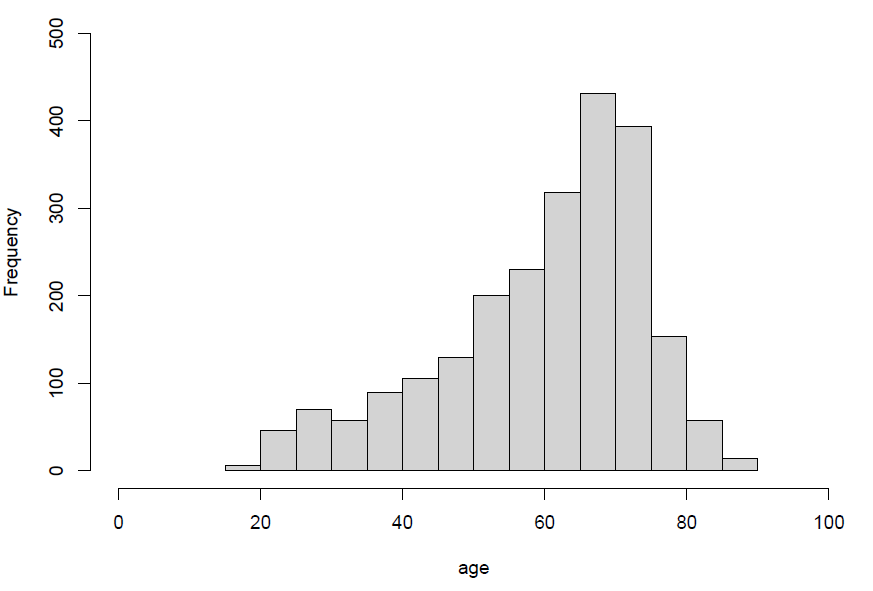


Fig A. The age distribution in the studied population


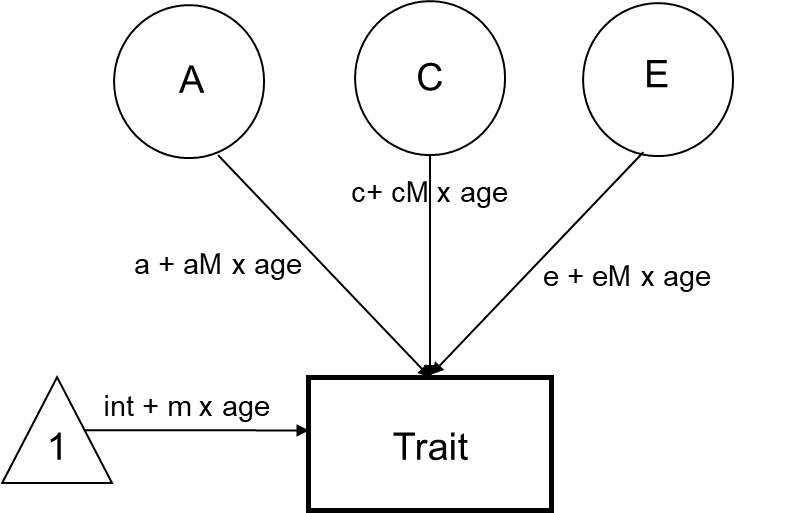


Fig B. Moderation models of genetic (A), shared environmental (C), and unique environmental (E) variances as a function of age. The moderation of age can occur on all three variances and as the main effect through a phenotypic regression.


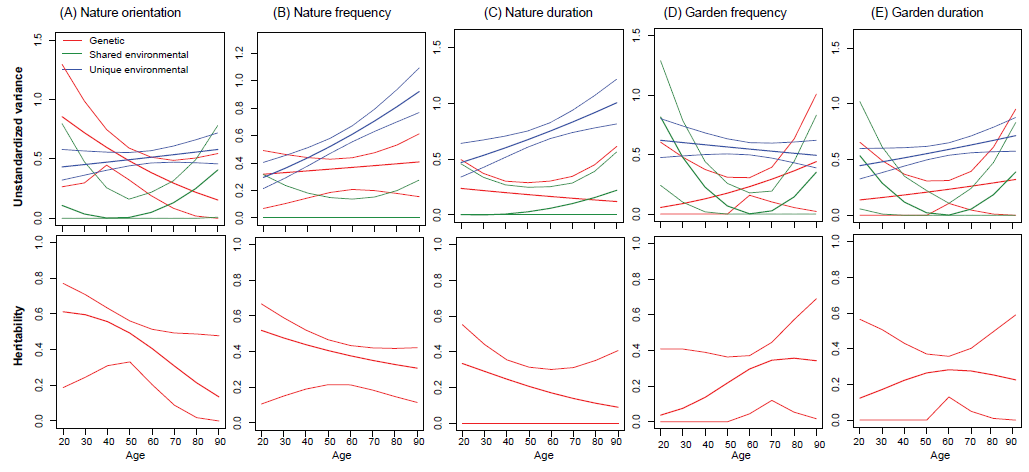


Fig C. Unstandardized genetic and environmental variances (upper panels) and heritability (lower panels) of the age moderation models controlling for sex in (A) nature orientation, (B) frequency of public nature space visits, (C) duration of public nature space visits, (D) frequency of garden visits, and (E) duration of garden visits as a function of age. Thick lines are the estimated variances across age, and thin lines are the 95% CIs.

Table A. Results of age moderation models, with controlling for sex, for nature orientation and each dimension of nature experience. Age is converted into age/100. Nature frequency = frequency of public nature space visits. Nature duration = duration of public nature space visits. Garden frequency = frequency of domestic garden visits. Garden duration = duration of domestic garden visits. The labels of path coefficient are shown in Fig B. Est = estimation.

|  | Orientation | | Nature frequency | | Nature duration | | Garden frequency | | Garden duration | |
| --- | --- | --- | --- | --- | --- | --- | --- | --- | --- | --- |
|  | Est | SE | Est | SE | Est | SE | Est | SE | Est | SE |
| a | 1.077 | 0.195 | 0.544 | 0.101 | 0.528 | 0.196 | 0.114 | 0.511 | 0.316 | 0.428 |
| c | -0.606 | 0.411 | <0.001 | 0.526 | -0.210 | 0.496 | 1.332 | 0.177 | 1.118 | 0.227 |
| e | 0.628 | 0.073 | 0.425 | 0.065 | 0.595 | 0.082 | 0.812 | 0.077 | 0.617 | 0.076 |
| aM | -0.760 | 0.409 | 0.106 | 0.174 | -0.201 | 0.504 | 0.612 | 0.815 | 0.279 | 0.733 |
| cM | 1.382 | 0.519 | <0.001 | 0.759 | 0.756 | 0.629 | -2.139 | 0.349 | -1.934 | 0.329 |
| eM | 0.148 | 0.122 | 0.595 | 0.113 | 0.454 | 0.141 | -0.121 | 0.125 | 0.254 | 0.129 |
| m | -0.516 | 0.190 | -0.589 | 0.159 | -0.120 | 0.158 | 2.324 | 0.179 | 2.103 | 0.171 |
| int | 0.317 | 0.120 | 0.353 | 0.097 | 0.074 | 0.096 | -1.386 | 0.112 | -1.257 | 0.105 |

Table B. Model comparisions between full moderation models and the models that dropped one moderation parameter. minus2LL = -2*Log-likelihood, df = degrees of freedom. AIC = Akaike information criterion. diffLL = difference in minus2LL. aM, cM, eM are the moderation effects on genetic, shared environmental and unique environmental influences.

| Nature orientation | |  |  |  |  |  |
| --- | --- | --- | --- | --- | --- | --- |
|  | comparison | minus2LL | df | AIC | diffLL | p |
| 1 | Full moderation | 6187.68 | 2260 | 1667.68 | NA | NA |
| 2 | Drop aM | 6189.99 | 2261 | 1667.99 | 2.32 | 0.128 |
| 3 | Drop cM | 6191.61 | 2261 | 1669.61 | 3.93 | 0.047 |
| 4 | Drop eM | 6189.08 | 2261 | 1667.08 | 1.40 | 0.237 |
| Nature frequency | |  |  |  |  |  |
|  | comparison | minus2LL | df | AIC | diffLL | p |
| 1 | Full moderation | 6316.30 | 2282 | 1752.30 | NA | NA |
| 2 | Drop aM | 6316.67 | 2283 | 1750.67 | 0.37 | 0.544 |
| 3 | Drop cM | 6316.30 | 2283 | 1750.30 | 0.00 | 1.000 |
| 4 | Drop eM | 6339.35 | 2283 | 1773.35 | 23.05 | <0.001 |
| Nature duration | |  |  |  |  |  |
|  | comparison | minus2LL | df | AIC | diffLL | p |
| 1 | Full moderation | 6419.66 | 2282 | 1855.66 | NA | NA |
| 2 | Drop aM | 6419.82 | 2283 | 1853.82 | 0.15 | 0.696 |
| 3 | Drop cM | 6420.21 | 2283 | 1854.21 | 0.54 | 0.461 |
| 4 | Drop eM | 6429.04 | 2283 | 1863.04 | 9.37 | 0.002 |
| Garden frequency | |  |  |  |  |  |
|  | comparison | minus2LL | df | AIC | diffLL | p |
| 1 | Full moderation | 6046.20 | 2280 | 1486.20 | NA | NA |
| 2 | Drop aM | 6046.84 | 2281 | 1484.84 | 0.64 | 0.423 |
| 3 | Drop cM | 6051.51 | 2281 | 1489.51 | 5.31 | 0.021 |
| 4 | Drop eM | 6047.15 | 2281 | 1485.15 | 0.95 | 0.330 |
| Garden duration | |  |  |  |  |  |
|  | comparison | minus2LL | df | AIC | diffLL | p |
| 1 | Full moderation | 6142.47 | 2280 | 1582.47 | NA | NA |
| 2 | Drop aM | 6142.63 | 2281 | 1580.63 | 0.15 | 0.697 |
| 3 | Drop cM | 6153.80 | 2281 | 1591.80 | 11.32 | 0.001 |
| 4 | Drop eM | 6146.21 | 2281 | 1584.21 | 3.73 | 0.053 |
