# Supplementary material for: People’s desire to be in nature and how they experience it are partially heritable
Source: PLoS Biol. 2022 Feb 3;20(2):e3001500. doi: 10.1371/journal.pbio.3001500 (PMC8812842; doi:10.1371/journal.pbio.3001500)
Supplement: S2 Note — (DOCX) [file pbio.3001500.s017.docx]

S2 Note. Questions and statements used to measure nature orientation and nature experiences

| Question | Value | Response | | | |
| --- | --- | --- | --- | --- | --- |
| In the spring and summer, how often do you usually visit public nature areas? | 0 | Never |  | | |
|  | 1 | Less than once every 2-3 months | | | |
|  | 2 | Once every 2-3 months | | | |
|  | 3 | Once a month | | | |
|  | 4 | 2-3 times a month | | | |
|  | 5 | Once a week | | | |
|  | 6 | 2-4 times a week | | | |
|  | 7 | 5-7 times a week | | | |
| In the spring and summer, when you visit a public nature area, how long do you usually spend there? | 1 | Up to 30 minutes | | | |
|  | 2 | >30 minutes to 1 hour | | | |
|  | 3 | >1 to 3 hours | | | |
|  | 4 | >3 to 5 hours | | | |
|  | 5 | >5 to 7 hours | | | |
|  | 6 | >7 to 9 hours | | | |
|  | 7 | >9 hours | | | |
| In the autumn and winter, how often do you usually visit public nature areas? | 0 | Never |  | | |
|  | 1 | Less than once every 2-3 months | | | |
|  | 2 | Once every 2-3 months | | | |
|  | 3 | Once a month | | | |
|  | 4 | 2-3 times a month | | | |
|  | 5 | Once a week | | | |
|  | 6 | 2-4 times a week | | | |
|  | 7 | 5-7 times a week | | | |
| In the autumn and winter, when you visit a public nature area, how long do you usually spend there? | 1 | Up to 30 minutes | | | |
|  | 2 | >30 minutes to 1 hour | | | |
|  | 3 | >1 to 3 hours | | | |
|  | 4 | >3 to 5 hours | | | |
|  | 5 | >5 to 7 hours | | | |
|  | 6 | >7 to 9 hours | | | |
|  | 7 | >9 hours | | | |
| Do you have a garden? | 1 | Yes | | | |
|  | 0 | No | | | |
| In spring and summer, how frequently do you usually spend more than 10 minutes in your garden? | 0 | Never | | | |
|  | 1 | Less than once a week | | | |
|  | 2 | 2-4 times a week | | | |
|  | 3 | 5-7 times a week | | | |
| In the spring and summer, whenever you spend time in your garden, how long do you usually spend? | 1 | Up to 30 minutes | | | |
|  | 2 | >30 minutes to 1 hour | | | |
|  | 3 | >1 to 3 hours | | | |
|  | 4 | >3 to 5 hours | | | |
|  | 5 | >5 to 7 hours | | | |
|  | 6 | >7 to 9 hours | | | |
|  | 7 | >9 hours | | | |
| In autumn and winter, how frequently do you usually spend more than 10 minutes in your garden? | 0 | Never | | | |
|  | 1 | Less than once a week | | | |
|  | 2 | 2-4 times a week | | | |
|  | 3 | 5-7 times a week | | | |
| In the autumn and winter, whenever you spend time in your garden, how long do you usually spend? | 1 | Up to 30 minutes | | | |
|  | 2 | >30 minutes to 1 hour | | | |
|  | 3 | >1 to 3 hours | | | |
|  | 4 | >3 to 5 hours | | | |
|  | 5 | >5 to 7 hours | | | |
|  | 6 | >7 to 9 hours | | | |
|  | 7 | >9 hours | | | |
| To what extent to do you agree with the following statements? Please provide an answer for each of the statements. | | | | | |
| The thought of being deep in the woods, away from civilization, is frightening. | 1 | Strongly disagree | | |  |
|  | 2 | Disagree | |  |  |
|  | 3 | Neutral | |  |  |
|  | 4 | Agree | |  |  |
|  | 5 | Strongly agree | | |  |
| My ideal vacation spot would be a remote, wilderness area. | 1 | Strongly disagree | | |  |
|  | 2 | Disagree | |  |  |
|  | 3 | Neutral | |  |  |
|  | 4 | Agree | |  |  |
|  | 5 | Strongly agree | | |  |
| I enjoy being outdoors, even in unpleasant weather. | 1 | Strongly disagree | | |  |
|  | 2 | Disagree | |  |  |
|  | 3 | Neutral | |  |  |
|  | 4 | Agree | |  |  |
|  | 5 | Strongly agree | | |  |
| I don’t often go out in nature. | 1 | Strongly disagree | | |  |
|  | 2 | Disagree | |  |  |
|  | 3 | Neutral | |  |  |
|  | 4 | Agree | |  |  |
|  | 5 | Strongly agree | | |  |
| I enjoy digging in the earth and getting dirt on my hands. | 1 | Strongly disagree | | |  |
|  | 2 | Disagree | |  |  |
|  | 3 | Neutral | |  |  |
|  | 4 | Agree | |  |  |
|  | 5 | Strongly agree | | |  |
| I take notice of wildlife wherever I am. | 1 | Strongly disagree | | |  |
|  | 2 | Disagree | |  |  |
|  | 3 | Neutral | |  |  |
|  | 4 | Agree | |  |  |
|  | 5 | Strongly agree | | |  |
